# Checklist of herpetofauna in the severely degraded ecosystem of Bidong Island, Peninsular Malaysia, South China Sea

**DOI:** 10.3897/zookeys.985.54737

**Published:** 2020-11-05

**Authors:** Muhamad Fatihah-Syafiq, Baizul Hafsyam Badli-Sham, Muhammad Fahmi-Ahmad, Mohamad Aqmal-Naser, Syed Ahmad Rizal, Mohd Shahrizan Azrul Azmi, Larry L. Grismer, Amirrudin B. Ahmad

**Affiliations:** 1 Faculty of Science and Marine Environment, Universiti Malaysia Terengganu, 21030 Kuala Nerus, Terengganu, Malaysia Universiti Malaysia Terengganu Kuala Nerus Malaysia; 2 Centre of Research and Field Service, Universiti Malaysia Terengganu, 21030 Kuala Nerus, Terengganu, Malaysia La Sierra University Riverside United States of America; 3 Herpetology Laboratory, Department of Biology, La Sierra University, Riverside, CA, USA Universiti Malaysia Terengganu Kuala Nerus Malaysia; 4 Institute of Tropical Biodiversity and Sustainable Development, Universiti Malaysia Terengganu, 21030 Kuala Nerus, Terengganu, Malaysia La Sierra University Riverside United States of America

**Keywords:** amphibian, Bidong Island, herpetological survey, Peninsular Malaysia, reptile, South China Sea

## Abstract

A herpetofaunal inventory was conducted on Bidong Island, Terengganu, Peninsular Malaysia. It incorporates data from a recent herpetological survey conducted from 1 to 3 April 2019 with reptile records from previous publications. Specimens were collected with drift-fenced pitfall traps and taxa were recorded with visual encounter surveys (VES). In total, 18 species of reptiles and amphibians were recorded, including three species of frogs, 12 species of lizards, and three species of snakes. Six species from the present survey are new records for the island.

## Introduction

The South China Sea has environmentally diverse groups of islands that have engaged researchers’ attention for the past two decades (Leong et al. 2003; Grismer et al. 2004; Grismer 2006; 2011a; 2011b). The three groups of islands located off the state of Terengganu are the Perhentian, Redang and Bidong Archipelagos. Faunal studies on these islands have indicated that these subregions support high herpetological diversity and endemism (Grismer et al. 2011). The islands of Perhentian and Redang have received extensive flora and fauna research in the past (e.g., Masayuki et al. 2007; Grismer and Chan 2008; Grismer et al. 2009, 2011; David et al. 2016; Hamza et al. 2016; Pesiu et al. 2016), but several islands in this area have been overlooked where it concerns terrestrial fauna. Tamblyn et al. (2005) reported on the herpetofaunal communities on Perhentian Kecil, Perhentian Besar and Redang islands. They reported 32 species of herpetofauna (three frogs, 21 lizards and eight snakes). The expeditions by Grismer and Chan (2008) and Grismer et al. (2009) to Perhentian Besar Island resulted in the descriptions of two new species: *Cnemaspisperhentianensis* and *Tytthoscincusperhentianesis*. Subsequently, Grismer et al. (2011) reported 46 species of herpetofauna from the Perhentian and Redang archipelagos, including Tenggol Island.

**Figure 1.**
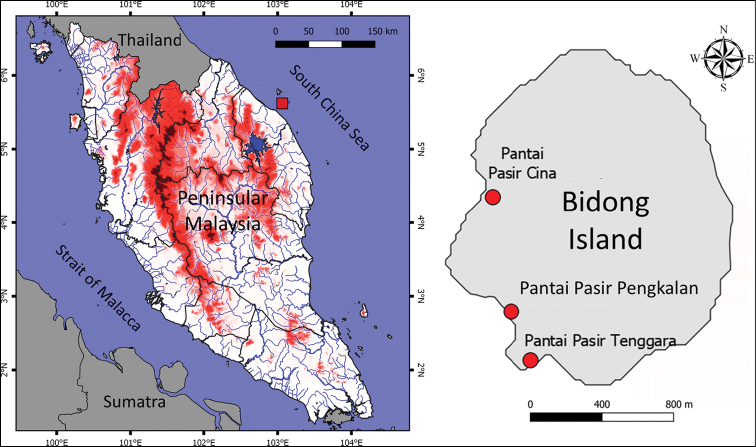
Map of Peninsular Malaysia (left) showing the location of Bidong Island, off the Terengganu coast, indicated by the red square. Map of Bidong Island (right) with the study locations indicated by red circles.

The Bidong Archipelago comprises six islands with Bidong Island (Fig. 1) being the largest. It is located about 33 km to the northeast of Kuala Terengganu. The islands of Karah, Gelok and Tengkorak are situated less than 5 km from Bidong Island, while about 15 km to the east lie Yu Kecil and Yu Besar Islands.

The fauna of Bidong Island was apparently first studied by Gibson-Hill (1952) who published a record of bird species. However, other terrestrial fauna was not reported until recently. Roslan et al. (2016) surveyed the island’s bat fauna, Fathihi-Hakimi et al. (2017) studied the butterfly diversity, and Grismer et al. (2014) described a new species of lizard, *Cnemaspisbidongensis*. Zakaria et al. (2017) followed up with an inventory of the island’s herpetofaunal diversity in which 12 species of lizards were recorded. Their study was a rapid survey made from 31 May to 7 June 2015 limited to the Pulau Bidong Marine Nature Research Station (**MNRS**) area near Pantai Pasir Cina.

In this article, we report the baseline data on a collection of amphibians and terrestrial reptiles acquired during a recent survey on the western side of Bidong Island in early April 2019. This report incorporates the findings of Zakaria et al. (2017) and presents an updated inventory of the herpetofaunal diversity of Bidong Island.

## Materials and methods

### Study area

Bidong Island, the largest island of the Bidong Archipelago, has a land area of about one square kilometre and is 321 m above sea level at its highest point. The island was used for housing Vietnamese refugees from 1975 to 1991. The settlement area (Fig. 1) is situated at Pantai Pasir Pengkalan, close to Pantai Pasir Tenggara. These two beaches can be accessed via a forest trail from the western part of the island where Pulau Bidong MNRS of Universiti Malaysia Terengganu is located at Pantai Pasir Cina. This island is covered with coastal and secondary forest composed of tree species such as *Terminalia catappa*, *Vaticacineria*, *Licaniasplendens* and *Hibiscustiliaceus* (Pesiu et al. 2016). The island’s landscape is hilly, dominated by extensive and steep granite outcrops with a few natural but seasonal drainages.

### Methods

Field surveys were conducted at the western part of Bidong Island, Terengganu, Malaysia from 1 to 3 April 2019. Study areas included chalets and research facilities, hilly areas, accessible forest trails, coastal vegetation as well as stagnant ponds near Pantai Pasir Cina (5°37'16"N, 103°3'28"E), Pantai Pasir Pengkalan (5°36'53"N, 103°3'32"E) and Pantai Pasir Tenggara (5°36'43"N, 103°3'36"E). Specimens were inventoried using the following approaches: Visual Encounter Survey (VES) and drift-fenced pitfall traps. VES is an active collecting method used to sample for species richness and abundance along the survey area by an observer under a time limit condition (Crump and Scott 1994). Drift-fenced pitfall trap is a passive collecting method for sampling ground dwelling amphibians and reptiles. Two sets of drift-fenced pitfall traps were deployed randomly about 200 m from MNRS and the Vietnamese settlement area. Each set of pitfall traps consisted of three buckets assembled in a straight line with fences measuring 0.5 m high and 4 m long. Each of the buckets used were punched with two or three drainage holes. These traps were opened for two consecutive days and checked before noon.

The VES method involved active searching and was conducted during the day (10 am to 4 pm) and at night (8 pm to 11 pm) to record both diurnal and nocturnal species. Animals were caught by hand or with snake tongs. Identification of amphibians follows Berry (1975) and the latest taxonomic nomenclature was used following Amphibian Species of the World database (Frost 2020). Identification of lizards follows Grismer (2006, 2011a, 2011b) and snakes follows Das (2010) and taxonomic nomenclature follows The Reptile Database (Uetz et al. 2020). All collected individuals were examined and a representative for each species was photographed in situ. Selected specimens were kept as vouchers to confirm the occurrence of their species on the island. Specimens were fixed with 10% formalin before being stored in a 70% ethanol solution. Samples of liver tissue were stored in 100% ethanol. All voucher specimens were deposited in the General Biology Lab., Universiti Malaysia Terengganu and catalogued under UMT Zoological Collection (**UMTZC**). Unpublished records of specimens in the General Biology Lab resulting from our pilot study in 2006 were also included to produce a composite herpetofaunal checklist. In addition, the record of reptile species reported by Zakaria et al. (2017) is presented in Table 1.

**Table 1.** List of terrestrial amphibians and reptiles recorded from Bidong Island, Terengganu in this study and from Zakaria et al. (2017).

Family	Species	Zakaria et al. (2017)	This study	IUCN Status
**Amphibians**				
Microhylidae	*Kaloulapulchra*	–	+	LC
	*Microhylaheymonsi*	–	+	LC
Rhacophoridae	*Polypedatesleucomystax*	–	+	LC
**Reptilians**				
Agamidae	*Bronchocelacristatella*	+	+	NE
Scincidae	*Dasiaolivacea*	+	+	LC
	*Eutropismultifasciata*	+	+	LC
Gekkonidae	*Cnemaspisbidongensis*	+	+	LC
	*Gekkocicakterbang*	+	+	NE
	*Gekkogecko*	+	+	LC
	*Gekkomonarchus*	+	–	NE
	*Hemidactylusfrenatus*	+	+	LC
	*Hemidactylusgarnotii*	+	–	NE
	*Hemidactylusplatyurus*	+	+	NE
	*Lepidodactyluslugubris*	+	+	NE
Varanidae	*Varanussalvator*	+	+	LC
Colubridae	*Lycodoncapucinus*	–	+	LC
Pythonidae	*Malayopythonreticulatus*	–	+	NE
Typhlopidae	*Indotyphlopsbraminus*	–	+	NE
Total no. of species/study		12	16	
Total no. of species		18		

In this checklist, taxonomic categories are alphabetically arranged and information for each species is presented in the following format: family name, species name, English name, notes and known distribution of the species in the offshore islands of Terengganu.

## Results

Table 1 incorporates data from this study and an earlier survey by Zakaria et al. (2017), and documents a total of 18 species of herpetofauna on Bidong Island (Table 1). These comprise three species of frogs from three genera belonging to the families Microhylidae (2 spp.) and Rhacophoridae (1 sp.); 12 species of lizards from nine genera belonging to the families Agamidae (1 sp.), Gekkonidae (8 spp.), Scincidae (2 spp.) and Varanidae (1 sp.); and three species of snakes from three genera belonging to the families Colubridae, Pythonidae and Typhlopidae with a single species in each family. The previous study by Zakaria et al. (2017) recorded two species of gekkonid lizards that were not found in the present survey. This study adds six new island records (3 amphibians and 3 snakes). Most of the herpetofauna species recorded from Bidong Island were either Least Concern (LC) or Not Evaluated (NE) according to the International Union for Conservation of Nature (**IUCN**) status (IUCN 2018).

### Class Amphibia

**Order Anura**

**Suborder Neobatrachia**

### Family Microhylidae

#### Kaloula pulchra

Taxon classificationAnimaliaAnuraMicrohylidae

Gray, 1831

FF101C3E-32DE-51A5-84BE-7FB156E55B7E

**Figure 2.**
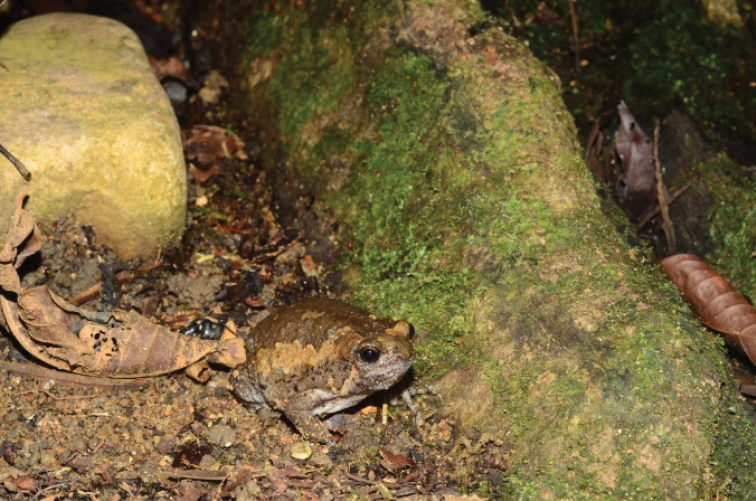
Kaloulapulchra

Banded bullfrog Fig. 2

##### Notes.

The banded bullfrog is fossorial but also shelters in burrows, tree holes and beneath surface objects. On 1 April 2019, one specimen was collected in a small tree cavity about 1.5 m above ground near MNRS at night.

##### Distribution.

This species is abundant in Peninsular Malaysia, and has also been found on Perhentian Besar, Redang and Tenggol islands (Grismer et al. 2011).

#### Microhyla heymonsi

Taxon classificationAnimaliaAnuraMicrohylidae

Vogt, 1911

AEFB258E-0D5F-5B6B-90FF-0F3005A77062

**Figure 3.**
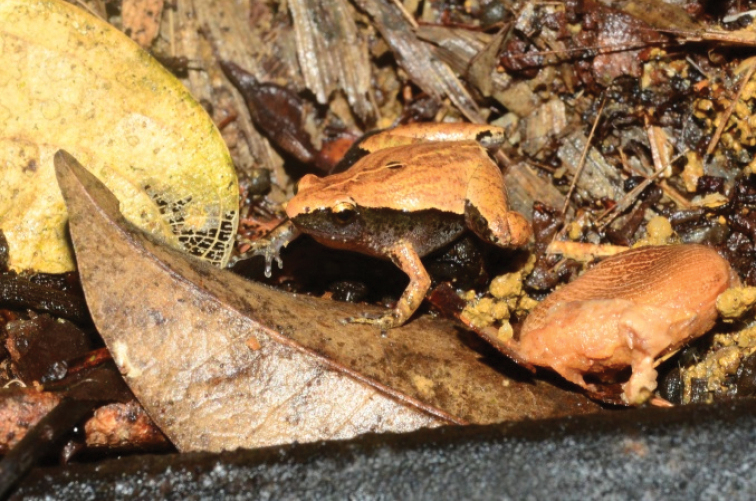
Microhylaheymonsi

Dark-sided chorus frog Fig. 3

##### Notes.

Many individuals were observed on the ground near puddles in grassy areas. Several males can be heard calling from the puddles. On 1 April 2019, a single specimen was collected from a stagnant puddle near MNRS at night. This specimen agrees with the morphology diagnosis by Garg et al. (2019).

##### Distribution.

This species is not known from other islands in Terengganu.

### Family Rhacophoridae

#### Polypedates leucomystax

Taxon classificationAnimaliaAnuraRhacophoridae

(Gravenhorst, 1829)

2AFEA38B-750D-5524-9303-AE753C961EA5

**Figure 4.**
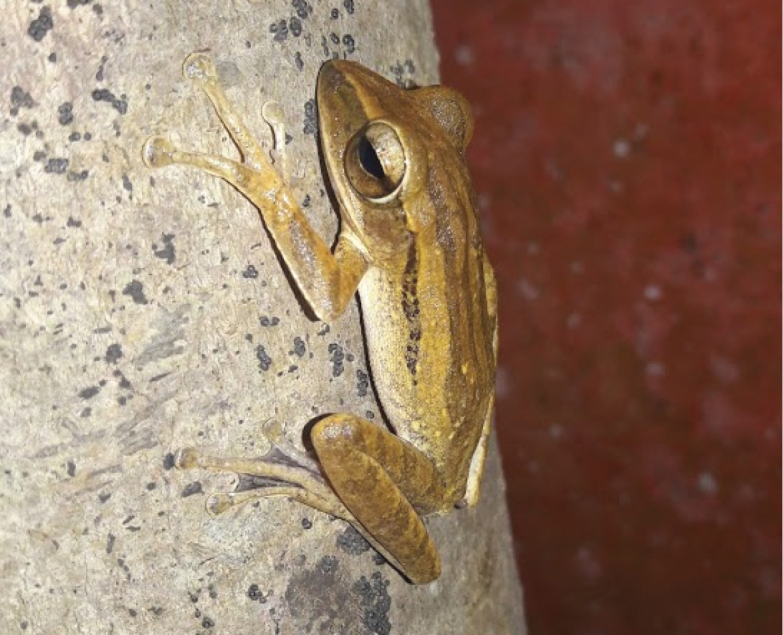
Polypedatesleucomystax

Four-lined tree frog Fig. 4

##### Notes.

This species was commonly observed on the hiking trail about 10 m above sea level. Individuals were located at night perching on leaves and branches of low to medium level vegetation. One was collected on a leaf 1 m above the ground at night on 2 April 2019. It matches the diagnosis of the species by Sumarli et al. (2015).

##### Distribution.

The four-lined tree frog is distributed throughout Perhentian Besar, Perhentian Kecil, Redang and Tenggol islands (Grismer et al. 2011).

### Class Reptilia

**Order Squamata**

**Suborder Sauria**

**Family Agamidae**

#### Bronchocela cristatella

Taxon classificationAnimaliaSquamataAgamidae

(Kuhl, 1820)

BF4D27DE-9C84-547A-84B3-E3EDD266D0B4

**Figure 5.**
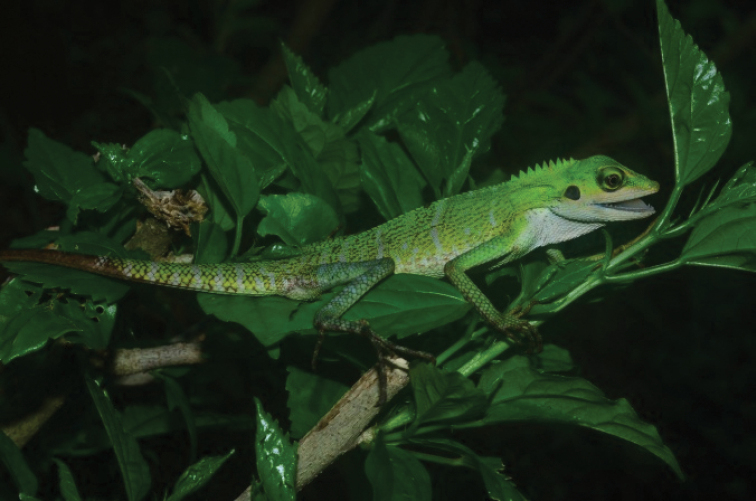
Bronchocelacristatella

Green crested lizard Fig. 5

##### Notes.

This striking green-coloured and diurnal lizard is active during the day and can be commonly found in open areas on bushes and trees from 1 to 3 m above the ground. On 2 April 2019, eight individuals were observed and a specimen was collected from Pantai Pasir Pengkalan. Two large individuals were seen climbing up to 3 m high on a tree near Pantai Pasir Tenggara . All examples on Bidong Island match the diagnosis of this species by Grismer (2011b).

##### Distribution.

This agamid also occurs on Perhentian Besar, Perhentian Kecil, Pulau Lang Tengah as well as Redang islands (Grismer 2011b; Grismer et al. 2011, 2015).

### Family Gekkonidae

#### Cnemaspis bidongensis

Taxon classificationAnimaliaSquamataGekkonidae

Grismer, Wood Jr., Ahmad, Sumarli, Vazquez, Ismail, Nance, Mohd-Amin, Othman, Rizaijessika, Kuss, Murdoch & Cobos, 2014

88A6E414-F222-5D4E-98BB-09D72F4B1006

**Figure 6.**
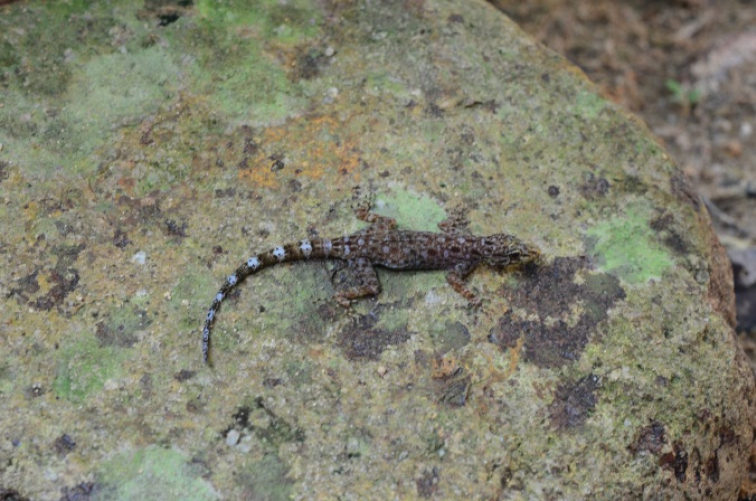
Cnemaspisbidongensis

Bidong island rock gecko Fig. 6

##### Notes.

*Cnemaspisbidongensis* is apparently endemic to Bidong Island where it seemed to be common. It inhabits forest in the interior of the island where it was found on rocks, twigs and tree trunks. About five individuals were observed on 1 April 2019 in the forest near MNRS. The species was described in 2014 by Grismer et al.

##### Distribution.

This gekkonid has thus far been found only on Bidong Island (Grismer et al. 2014).

#### Gekko cicakterbang

Taxon classificationAnimaliaSquamataGekkonidae

(Grismer, Wood Jr., Grismer, Quah, Thy, Phimmachak, Sivongxay, Seateun, Stuart, Siler, Mulcahy, Anamza & Brown, 2019)

8AA727C2-1D88-5A19-B38B-E3F4505EBC51

**Figure 7.**
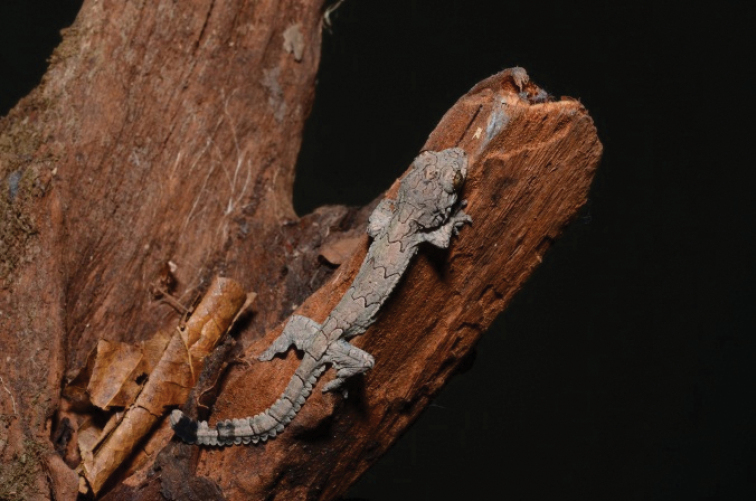
Gekkocicakterbang

Malaysian parachute gecko Fig. 7

##### Notes.

This gecko was occasionally observed on man-made structures in MNRS. It is nocturnal and apparently has the capability to glide from one tree to another. Until 2019, this species was thought to be conspecific with *Ptychozoonlionotum* (see Grismer et al. 2019 as *Ptychozooncicakterbang*). Ptychozoonlater became a subgenus of Gekko (see Wood et al. 2020).

##### Distribution.

*Gekkocicakterbang* ranges throughout Peninsular Malaysia and its associated islands but in the islands off Terengganu, it has only been found on Perhentian Besar, Redang, and Bidong islands (Grismer 2011b; Grismer et al. 2011, 2019).

#### Gekko gecko

Taxon classificationAnimaliaSquamataGekkonidae

(Linnaeus, 1758)

7C0A5578-D8CE-50C1-8F61-7EE561FE03D1

Tokay gecko Fig. 8

##### Notes.

This large species of gecko was common on Bidong Island, especially at the MNRS area. Individuals observed match the diagnosis of the species by Grismer (2011b).

##### Distribution.

The Tokay Gecko has also been recorded from Perhentian Besar, Perhentian Kecil and Redang islands (Grismer 2011b; Grismer et al. 2011).

**Figure 8.**
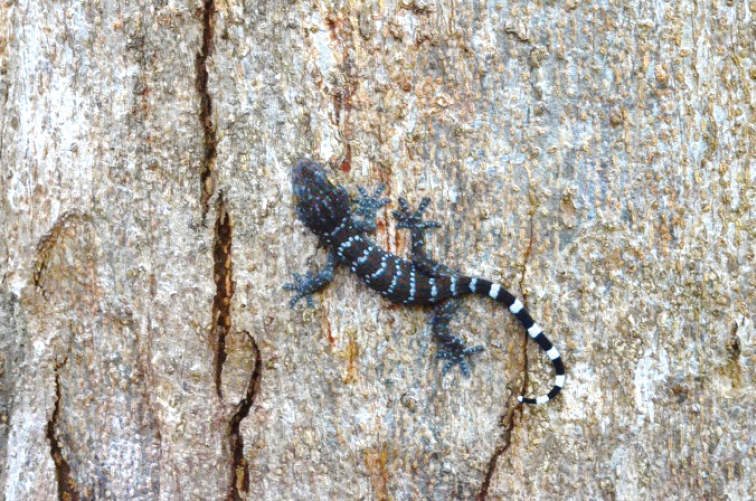
Gekkogecko

#### Gekko monarchus

Taxon classificationAnimaliaSquamataGekkonidae

(Schlegel, 1836)

A4585FDA-8C65-55FF-9B30-284C78D012CD

##### Notes.

This species was not sighted in this study but was reported by Zakaria et al. (2017).

##### Distribution.

*Gekkomonarchus* is known from Perhentian Besar, Redang and Tenggol islands (Grismer 2011b; Grismer et al. 2011).

#### Hemidactylus frenatus

Taxon classificationAnimaliaSquamataGekkonidae

Schlegel, 1836

655CB2FF-5F3D-5CCD-9F54-52A62617ECBB

##### Notes.

*Hemidactylusfrenatus* is a widespread species that seems to have a preference for human settlements and disturbed forest on Bidong Island. Individuals observed match the diagnosis of this species in Grismer (2011b).

##### Distribution.

This gekkonid is found on all of Terengganu’s offshore islands except Susu Dara Kecil Island (Grismer 2011b; Grismer et al. 2011).

#### Hemidactylus garnotii

Taxon classificationAnimaliaSquamataGekkonidae

Duméril & Bibron, 1836

E49E1576-33D7-519D-9B96-36CF3C0A04BC

##### Notes.

Although reported to occur on Bidong Island by Zakaria et al. (2017), none was observed in the recent study.

##### Distribution.

This species has been observed in Kuala Aring, Kelantan (Grismer 2011b) and Karah Island near Bidong Island (Grismer, unpublished data).

#### Hemidactylus platyurus

Taxon classificationAnimaliaSquamataGekkonidae

(Schneider, 1797)

85A97CBC-68AE-54B4-87D8-0834CC2C2C80

##### Notes.

This species was observed living in syntopy with *Hemidactylusfrenatus* in human settlements near MNRS. Individuals seen match the diagnosis of this species in Grismer (2011b).

##### Distribution.

Off the Terengganu coast, Perhentian Besar Island seems to be the only other island where *Hemidactylusplatyurus* has been recorded (Grismer 2011b; Grismer et al. 2011).

#### Lepidodactylus lugubris

Taxon classificationAnimaliaSquamataGekkonidae

(Duméril & Bibron, 1836)

6C40F84A-D7F4-5501-8C73-6F70A5717B0F

**Figure 9.**
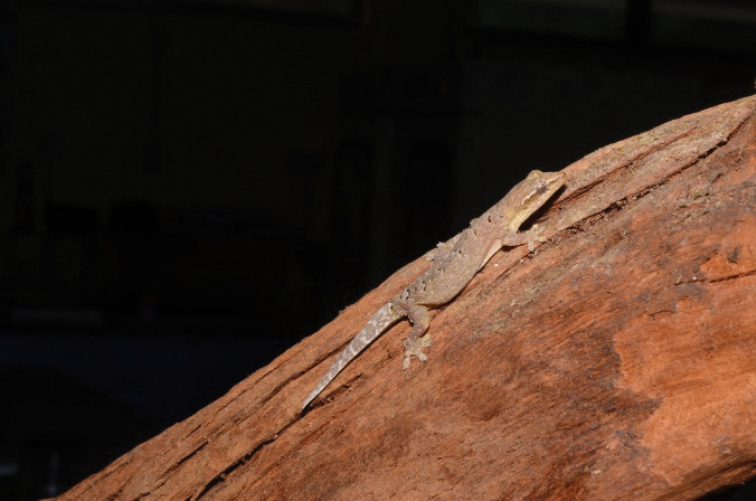
Lepidodactyluslugubris

Mourning gecko Fig. 9

##### Notes.

On Bidong Island, one individual was found near Pantai Pasir Pengkalan. Its appearance matches the diagnosis of the species in Grismer (2011b).

##### Distribution.

This species has also been recorded from Susu Dara Kecil Island (Grismer 2011b; Grismer et al. 2011).

### Famil Scincidae

#### Squamata olivacea

Taxon classificationAnimaliaSquamataScincidae

Gray, 1839

334BA900-64EF-5239-9B46-73C119FFFC3D

##### Notes.

*Dasiaolivacea* is diurnal, arboreal species that can be seen basking head down on tree trunks. About three individuals were observed from 1 to 3 April 2019, and all were about 2 m high on trees. One was seen with a small, unidentified gecko in its mouth on 2 April 2019. The predation of this species on geckos was reported by Grismer (2011b) on Babi Tengah Island in Johor. Grismer (2011b) observed that this skink also feeds on large centipedes, which suggests that *Dasiaolivacea* is an opportunistic feeder that does not have a specialized diet. The morphology and colour pattern of the individuals observed on Bidong Island match the diagnosis of the species by Grismer (2011b).

##### Distribution.

This species is also recorded from Perhentian Besar, Perhentian Kecil, Redang and Tenggol islands (Grismer 2011b; Grismer et al. 2011).

#### Eutropis multifasciata

Taxon classificationAnimaliaSquamataScincidae

(Kuhl, 1820)

132C20D6-6E5B-5C56-815F-0249A25B88E1

**Figure 10.**
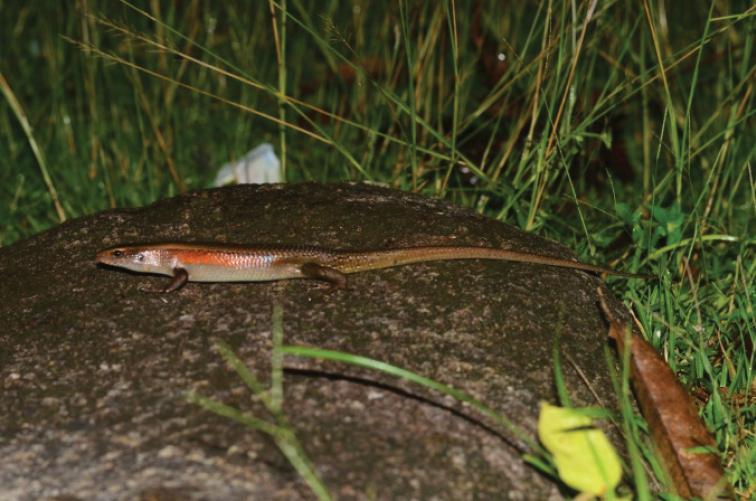
Eutropismultifasciata

Javan sun skink Fig. 10

##### Notes.

This common skink can be found across almost all habitats, including disturbed forest, open areas, human settlements and primary forest. It was very common at Pantai Pasir Pengkalan with 11 individuals being recorded during the recent survey. Specimens observed match the diagnosis of this species by Grismer (2011b).

##### Distribution.

This skink has also been reported from Perhentian Besar, Redang, and Tenggol islands (Grismer 2011b; Grismer et al. 2011).

### Famil Varanidae

#### Varanus salvator

Taxon classificationAnimaliaSquamataVaranidae

(Laurenti, 1768)

91FFFBB7-BE67-5D86-B477-169A9C36FE96

##### Notes.

Several individuals of this large, diurnal lizard that weres seen frequently on Bidong Island from 1 to 3 April 2019, especially near MNRS, due to the availability of food at the garbage piles. They were observed foraging in the afternoon and evening before dusk. The appearance of those lizards matches the diagnosis of this species in Grismer (2011b).

##### Distribution.

This species has been recorded on many islands off Terengganu except Susu Dara Kecil, Seringgeh and Tokong Burung Besar islands (Grismer 2011b; Grismer et al. 2011).

### Suborder Serpentes

**Family Colubridae**

#### Lycodon capucinus

Taxon classificationAnimaliaSquamataColubridae

(Boie, 1827)

06714997-C4A3-542F-83D1-7545F158EAE3

**Figure 11.**
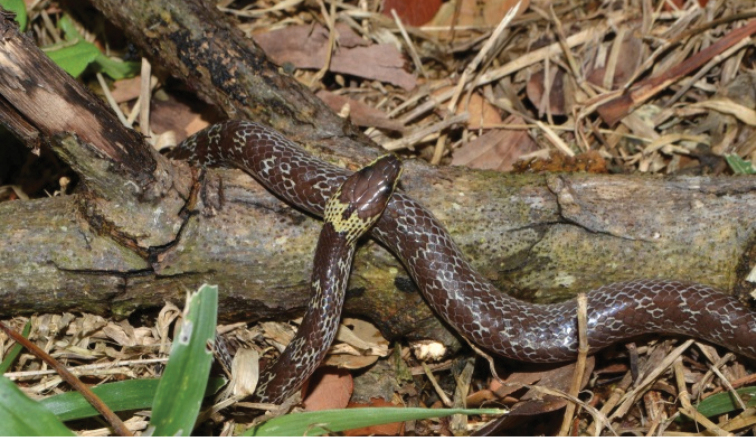
Lycodoncapucinus

Common wolf snake Fig. 11

##### Notes.

This nocturnal snake was common on Bidong Island where individuals were often found beneath logs, rocks and beach debris. Several individuals were observed near MNRS and Pantai Pasir Pengkalan on 1and 2 April 2019. The individuals examined match the diagnosis of the species in Grismer et al. (2011).

##### Distribution.

Also recorded from Perhentian Besar Island (Grismer et al. 2011).

### Family Pythonidae

#### Malayopython reticulatus

Taxon classificationAnimaliaSquamataPythonidae

(Schneider, 1801)

DD1248FB-CC71-5E13-A753-1BC095D556F5

##### Notes.

This large and widespread snake can be found in a wide variety of habitats and preys on large animals. A 2 m long individual was spotted at night on 2 April 2019 among grass near Pantai Pasir Pengkalan. It fits the diagnosis of the species in Grismer (2006).

##### Distribution.

Also recorded from Perhentian Besar, Susu Dara Besar, Redang and Tenggol islands (Grismer et al. 2011).

### Family Typhlopidae

#### Indotyphlops braminus

Taxon classificationAnimaliaSquamataTyphlopidae

(Daudin, 1803)

01441C8B-30C4-57CB-919C-F4B12798A880

##### Notes.

This small fossorial snake resembles an earthworm and seems to occur largely in human settlements. It feeds on the larvae of ants and termites, and is known to be parthenogenic. One specimen was captured on surface debris at night on 2 April 2019 near MNRS. Its morphology matches the diagnosis of the species in Grismer (2006).

##### Distribution.

*Indotyphlopsbraminus* was recorded by Grismer et al. (2011) from Perhentian Besar Island.

## Discussion

Human activities are the major cause of an island’s ecosystem degradation around the world (see Graham et al. 2017) and Terengganu’s offshore islands are no exception. The continuous encroachment is perturbing, as anthropogenic disturbances are detrimental to terrestrial insular species (Trainor 2007). Habitat modification is one of the human activities that drive herpetofaunal species into extirpations particularly for habitat specialist species (Gibbons et al. 2000; Kanowski et al. 2006). Bidong Island has been greatly degraded by the establishment of the refugee camps from 1975 to 1991 which likely threatened its terrestrial fauna. About three quarter of the forested areas on the island has been disturbed during the Vietnamese occupancy. The natural vegetation left can be mostly found on the north-eastern and northern sides on the island. However the endemic gecko, *Cnemaspisbidongensis*, did not seem affected. Grismer et al. (2014) inferred that *C.bidongensis*, unlike most of its congeners, is not a microhabitat specialist. This could have helped its survival undeterred by the island’s severely degraded ecosystem in the past. Persistence and recovery of fauna to overcome disturbances has been studied in many habitats (Gardner et al. 2007; Dent and Wright 2009; Sayer et al. 2017) but in islands this has been less addressed, particularly in Peninsular Malaysia.

It has been almost 30 years since the Vietnamese occupants left Bidong Island and the persistence of the herpetofauna to withstand the human-imposed pressure in the past is remarkable despite the great deterioration of the island’s forest ecosystem. Since then, anthropogenic abandonment and forest succession may help terrestrial herpetofauna to recover (Hilje and Aide 2012; Hernandez-Ordonez et al. 2015), even more rapidly on the island (Ríos-López and Aide 2007; Herrera-Montes and Brokaw 2010; Acevedo-Charry and Aide 2019). The findings in this study have demonstrated the resiliency and persistence of this island’s herpetofauna over the four years separating the two reported inventories. Yet, further tests with extensive monitoring data are required to elucidate the shift in the community structure. Previous records of a reptile inventory in 2015 documented a total number of 12 species (Zakaria et al. 2017) and this study managed to confirm ten species out of that number. This study however, augmented the herpetofaunal study in this island by including amphibians and also adding three new records of reptiles making a total of 18 herpetofauna species for Bidong Island.

Many of the species on the island are to some extent human commensals, and some may even have been introduced with human occupation of the island. High densities of species such as *Microhylaheymonsi*, *Kaloulapulchra*, *Gekkogecko*, *Varanussalvator*, *Hemidactylusfrenatus* and *Bronchocelacristatella* were also observed in this study. Anthropogenic-adapated species such as *G.gecko* and *H.frenatus* could also be accidentally introduced by humans during the arrival of the refugees on the island, which also may influence the abundance of these species on this island. Hypothetically, the abundance of these species may result from the availability of microhabitat as refugia, lack of predators, availability of food resources, human introduction and density compensation due to missing interspecific competitors in the island (Stamps and Buechner 1985; Ernst et al. 2006; Hilje and Aide 2012; Novoslov et al. 2016). Ephemeral habitats such as temporary ponds help generalist amphibians like *M.heymonsi* to thrive in harsh conditions. These ponds can be the source for natal habitat or shelters for amphibian species to recolonize back after the successional stage (Pittman et al. 2014). Lack of predation pressure also can induce the colonization of the frogs and lizards. To date, only three species of snakes are known from the island namely *Indotyphlopsbraminus*, *Lycodoncapucinus* and *Malayopythonreticulatus*. Of these, *L.capucinus* is highly likely the only predator of the frogs and lizards but again, sampling effort plays an important role to deliver accurate information on true species richness of snakes in this island because snakes are elusive. Zakaria et al. (2017) were unable to record any snakes and these new records are attributed to expanded survey areas, additional co-workers, and different sampling months.

Generally, many species that occur on this island are ubiquitous species; hence these do not require a specialized microhabitat. Reptiles in particular, have a suit of biological traits that may help them to subsist on this island such as efficiency in energy expenditure, flexibility in growth development and ability to shift diet spatially and/or temporally (see Shine and Somaweera 2019). These adaptations perhaps are the reasons for their persistence, but further studies are needed to test this. In addition, with the exception of *M.reticulatus* and *I.braminus*, large numbers of individuals of other species in Pantai Pasir Pengkalan and Pantai Pasir Cina indicated that they can be expected to be widespread throughout the island. Hence, most of these species are listed as LC in IUCN status as they are also ubiquitous on the mainland and several other islands in Terengganu.

The results of this inventory are not conclusive, as the studies done only covered several small sites of the island. Large parts of the island forest are still unexplored, thus the results may not represent the true species richness of the island. The north-eastern part of the island is rugged with steep slopes and sharp outcrops, hence has limited access during the survey. The northern, central and eastern parts of the islands’ forests are still unexplored. These large areas (which encompassed 60% of the island’s landscape) are now covered by mature secondary forest and receive less human disturbance after the recovery of the forest from the past incident. Increased sampling efforts, in terms of sampling areas and sampling time, may result in the discovery of new island records, perhaps even new ground dwelling species with the installation of pitfall traps. Long-term monitoring and ecological studies are needed to realize the true diversity of herpetofauna and improve the conservation of the terrestrial herpetofauna.The herpetofauna of Bidong Island survived the catastrophic ecological event in the past, but the resilience of species to recover after land abandonment and forest succession (true species richness and population density) remains in question. Abandoned habitat from the past and intact forests need to be maintained and undisturbed to promote the persistence of this island’s herpetofauna. This study provides baseline data for ecological monitoring and studies which are crucial to uncover the diversity of this insular herpetofauna and inform conservation authorities and stakeholders about current and future conservation management.

## Supplementary Material

XML Treatment for Kaloula pulchra

XML Treatment for Microhyla heymonsi

XML Treatment for Polypedates leucomystax

XML Treatment for Bronchocela cristatella

XML Treatment for Cnemaspis bidongensis

XML Treatment for Gekko cicakterbang

XML Treatment for Gekko gecko

XML Treatment for Gekko monarchus

XML Treatment for Hemidactylus frenatus

XML Treatment for Hemidactylus garnotii

XML Treatment for Hemidactylus platyurus

XML Treatment for Lepidodactylus lugubris

XML Treatment for Squamata olivacea

XML Treatment for Eutropis multifasciata

XML Treatment for Varanus salvator

XML Treatment for Lycodon capucinus

XML Treatment for Malayopython reticulatus

XML Treatment for Indotyphlops braminus

## References

[B1] Acevedo-Charry O Aide TM (2019) Recovery of amphibian, reptile, bird and mammal diversity during secondary forest succession in the tropics. Oikos 128: 1065–1078. 10.1111/oik.06252

[B2] Berry PY (1975) The amphibian fauna of Peninsular Malaysia. Tropical Press, Kuala Lumpur, 130 pp.

[B3] Crump ML Scott NJ (1994) Visual encounter surveys. In: Heyer WR Donnelley MA McDiarmid RW Hayek LAC Foster MS (Eds) Measuring and monitoring biological diversity: Standard methods for amphibians.Smithsonian Institution Press, Washington, 84–92.

[B4] Das I (2010) A field guide to the Reptiles of South-East Asia. New Holland Publishers (UK) Ltd, London–Cape Town–Sydney–Auckland, 376 pp.

[B5] David G Roslan A Mamat MA Abdullah MT Hamza AA (2016) A brief survey on birds from Pulau Perhentian Besar, Terengganu. Journal of Sustainability Science and Management (Special Issue Number 1), The International Seminar on the Straits of Malacca and the South China Sea 2016: 11–18.

[B6] Dent D Wright SJ (2009) The future of tropical species in secondary forests: A quantitative review. Biological Conservation 142: 2833–2843. 10.1016/j.biocon.2009.05.035

[B7] Ernst R Linsemair KE Mark-Oliver R (2006) Diversity erosion beyond the species level: Dramatic loss of functional diversity after selective logging in two tropical amphibian communities. Biological Conservation 133: 143–155. 10.1016/j.biocon.2006.05.028

[B8] Fathihi-Hakimi R Muhamad-Aidil Z Adanan A Azizah AA Pesiu E Abdullah MT (2017) Checklist of butterflies in Pulau Perhentian and Pulau Bidong, Terengganu. Journal of Sustainability Science and Management 12: 40–48.

[B9] Frost DR (2020) Amphibian Species of the World: an Online Reference. Version 6.1. http://research.amnh.org/herpetology/amphibia/index.html [accessed on 9 July 2020]

[B10] Gardner TA Ribeiro-Junior MA Barlow J Avila-Peres TCS Hoogmoed MS Peres CA (2007) The value of primary, secondary, and plantation forests for a Neotropical herpetofauna. Conservation Biology 21: 775–787. 10.1111/j.1523-1739.2007.00659.x 17531055

[B11] Garg S Suyesh R Das A Jiang J Wijayathilaka N Amarasinghe AAT Alhadi F Vineeth KK Aravind NA Senevirathne G Meegaskumbura M Biju SD (2019) Systematic revision of *Microhyla* (Microhylidae) frogs of South Asia: a molecular, morphological, and acoustic assessment. Vertebrate Zoology 69: 1–71.

[B12] Gibbons JW Scott DE Ryan TJ Buhlmann KA Tuberville TD Metts BS Greene JL Mills T Leiden Y Poppy S Winne CT (2000) The global decline of reptiles, deja vu amphibians. BioScience 50: 653–666. 10.1641/0006-3568(2000)050[0653:TGDORD]2.0.CO;2

[B13] Gibson-Hill CA (1952) Ornithological notes from the Raffles Museum 15, notes on the avifauna of great Redang Island (Terengganu). Bulletin of Raffles Museum 24: 220–240.

[B14] Graham NR Gruner DS Lim JY Gillespie RG (2017) Island ecology and evolution: Challenges in the Anthropocene. Environmental Conservation 44: 323–335. 10.1017/S0376892917000315

[B15] Grismer JL Grismer LL Das I Yaakob NS Liat LB Leong TM Youmans TM Kaiser H (2004) Species diversity and checklist of the herpetofauna of Pulau Tioman, Peninsular Malaysia with a preliminary overview of habitat utilization. Asiatic Herpetological Research 10: 244–276.

[B16] Grismer LL (2006) Amphibians and reptiles of the Tioman Archipelago, Malaysia. Forestry Department of Peninsular Malaysia, Kuala Lumpur, 216 pp.

[B17] Grismer LL (2011a) Amphibians and reptiles of the Seribuat Archipelago (Peninsular Malaysia). Edition Chimaira, Frankfurt am Main, 239 pp.

[B18] Grismer LL (2011b) Lizards of Peninsular Malaysia, Singapore, and their adjacent archipelagos: Their description, distribution, and natural history. Edition Chimaira, Frankfurt am Main, 728 pp.

[B19] Grismer LL Chan KO (2008) A new species of *Cnemaspis* Strauch 1887 (Squamata: Gekkonidae) from Pulau Perhentian Besar, Terengganu, Peninsular Malaysia. Zootaxa 1771: 1–15. 10.11646/zootaxa.1771.1.1

[B20] Grismer LL Grismer JL Quah ESH Thy N Phimmachak S Sivongxay N Seateun S Stuart BL Siler CB Mulcahy DG Anamza T Brown RM (2019) Geographic structure of genetic variation in the Parachute Gecko *Ptychozoonlionotum* Annandale, 1905 across Indochina and Sundaland with descriptions of three new species. Zootaxa 4638: 151–198. 10.11646/zootaxa.4638.2.1 31712473

[B21] Grismer LL Grismer JL Wood Jr PL Ngo VT Neang T Chan KO (2011) Herpetology on the fringes of the Sunda Shelf: A discussion of discovery, taxonomy, and biogeography. Bonner Zoologische Monographien 57: 57–97.

[B22] Grismer LL Wood Jr PL Ahmad AB Sumarli ASI Vazquez JJ Ismail LHB Nance R Mohd-Amin MAB Othman MNAB Rizal SA Kuss M Murdoch M Cobos A (2014) A new species of insular rock gecko (Genus *Cnemaspis* Strauch, 1887) from the Bidong Archipelago, Terengganu, Peninsular Malaysia. Zootaxa 3755: 447–456. 10.11646/zootaxa.3755.5.4 24869832

[B23] Grismer LL Wood Jr PL Grismer JL (2009) A new insular skink of the genus *Sphenomorpus* Strauch 1887 (Squamata: Scincidae) from Pulau Perhentian Besar, Terengganu, Peninsular Malaysia. Tropical Life Sciences Research 20: 51–69. 10.11646/zootaxa.1771.1.1

[B24] Grismer LL Wood Jr PL Lee CH Quah ESH Anuar S Ngadi E Sites Jr JW (2015) An integrative taxonomic review of the agamid genus *Bronchocela* (Kuhl, 1820) from Peninsular Malaysia with descriptions of new montane and insular endemics. Zootaxa 3948: 1–23. 10.11646/zootaxa.3948.1.1 25947760

[B25] Hamza AA Wong CH Ahmad A (2016) Rediscovery of least known breeding sites for seabirds in East Coast Peninsular Malaysia. Malayan Nature Journal 68: 121–129.

[B26] Hernandez-Ordonez O Urbina-Cardona N Martinez-Ramos M (2015) Recovery of amphibian and reptile assemblages during old-field succesion in tropical rain forests. Biotropica 46: 377–388. 10.1111/btp.12207

[B27] Herrera-Montes A Brokaw N (2010) Conservation value of tropical secondary forest: A herpetofaunal perspective. Biological Conservation 143: 1414–1422. 10.1016/j.biocon.2010.03.016

[B28] Hilje B Aide TM (2012) Recovery of amphibian species richness and composition in a chronosequence of secondary forests, northeastern Costa Rica. Biological Conservation 146: 170–176. 10.1016/j.biocon.2011.12.007

[B29] IUCN (2018) IUCN Red List Categories and Criteria. Version 3.1. International Union for Conservation of Nature and Natural Resources, Gland.

[B30] Kanowski JJ Reis TM Catterall CP Piper SD (2006) Factors affecting the use of reforested sites by reptiles in cleared rainforest landscapes in tropical and subtropical Australia. Restoration Ecology 14: 67–76. 10.1111/j.1526-100X.2006.00106.x

[B31] Leong TM Grismer LL (2003) Preliminary checklists of the Anambas and Natuna Islands (South China Sea). Hamadryad 27: 165–174.

[B32] Masayuki M Heng C Ahmad A (2007) A survey of ant species at Chagar Hutang, Redang Island and a new record of *Anoplolepisgracilipes*, an invasive species. Malayan Nature Journal 59: 205–211.

[B33] Novoslov M Rodda GH Feldman A Kadison AE Dor R Meiri S (2016) Power in numbers: Drivers of high population density in insular lizards. Global Ecology and Biogeography 25: 87–95. 10.1111/geb.12390

[B34] Pesiu E Abdullah MT Salim J Salam MR (2016) Tree Species composition in Pulau Bidong and Pulau Redang. Journal of Sustainability Science and Management (Special Issue Number 1), The International Seminar on the Straits of Malacca and the South China Sea 2016: 48–60.

[B35] Pittman SE Osbourn MS Semlitsch RD (2014) Movement ecology of amphibians: A missing component for understanding population declines. Biological Conservation 169: 44–53. 10.1016/j.biocon.2013.10.020

[B36] Ríos-López N Aide TM (2007) Herpetofaunal dynamics during secondary succession. Herpetologica 63: 35–50. 10.1655/0018-0831(2007)63[35:HDDSS]2.0.CO;2

[B37] Roslan A David G Nur-Izzah IA Rahim NAA Pesiu E Muhamad-Aidil Z Fathihi-Hakimi R Hasrulzaman H Mohamad-Abid K Mohamed NZ Abdullah MT (2016) Notes of bats in Pulau Bidong and Pulau Perhentian Besar, Terengganu, Malaysia. Journal of Sustainability Science and Management (Special Issue Number 1), The International Seminar on the Straits of Malacca and the South China Sea 2016: 26–35.

[B38] Sayer CA Bullock JM Martin P (2017) Dynamics of avian species and functional diversity in secondary tropical forests. Biological Conservation 211: 1–9. 10.1016/j.biocon.2017.05.004

[B39] Shine R Somaweera R (2019) Last lizard standing: The enigmatic persistence of the komodo dragon. Global Ecology and Conservation 18: e00624. 10.1016/j.gecco.2019.e00624

[B40] Stamps JA Buechner M (1985) The territorial defense hypothesis and the ecology of insular vertebrates. The Quaterly Review of Biology 60: 155–181. 10.1086/414314 3895283

[B41] Sumarli AX Grismer LL Anuar S Muin MA Quah ESH (2015) First report on the amphibians and reptiles of a remote mountain, Gunung Tebu in northeastern Peninsular Malaysia. CheckList 11: 1–32. 10.15560/11.4.1679

[B42] Tamblyn AN Turner C O’Mally R Weaver N Hughes T Hardingham S Roberts H (2005) Malaysia Tropical Forest Conservation Project Report of the Perhentian Phase 2005. Coral Cay Conservation Ltd., London, 105 pp.

[B43] Trainor CR (2007) Changes in bird species compositions on a remote and well forested Wallacean island, South East Asia. Biological Conservation 140: 373–385. 10.1016/j.biocon.2007.08.022

[B44] Uetz P Freed P Hosek J (2020) The Reptile Database. http://www.reptile-database.org [accessed on 9 July 2020]

[B45] Wood Jr PL Guo X Travers SL Yong-Chao S Olson KV Bauer AM Grismer LL Siler CD Moyle RG Andersen MJ Brown RM (2020) Parachute geckos free fall into synonymy: *Gekko* phylogeny, and a new subgeneric classification, inferred from thousands of ultraconserved elements. Molecular Phylogenetic and Evolution 146: 106731. 10.1016/j.ympev.2020.106731 31904508

[B46] Zakaria AA Rahim NAA Abdullah MT (2017) Reptile diversity as an ecotourism attraction in Pulau Bidong. In: Mariapan M Lin ELA Isa SS Karim MS Hakeem KR (Eds) Ecotourism potentials in Malaysia.Faculty of Forestry Universiti Putra Malaysia, Serdang, Selangor, 42–47.

